# Surgery

**Published:** 1879-02

**Authors:** 


*Collaborators:*

DR. H. GRADLE, DR. L. W. CASE, DR. R. PARK,
DR. R. TILLEY, DR. D. R. BROWER.

### SURGERY.

WOUND OF PLEURA AND LUNG TISSUE—LAPAROTOMY TO REDUCE HERNIA. (*Journal de Médecine et de Chirurgie pratiques.*) Hôpital de Bicêtre. Service de M. le Docteur F. Terrier.

X, aged 65, in a struggle received a knife wound in the third intercostal space external to the mamary glands. No pain was experienced at the moment when the wound was inflicted, the man not being conscious of the state until he saw the blood.

On examination he was found to be breathing through the wound. This was demonstrated by a prolonged whistling sound perceived at each respiration, and at each expiration by the flow of arterial blood mixed with large bubbles of air. The wound was two and one-half centimeters in length, and somewhat ragged. The bronchial tubes could not have been much wounded because he vomited no blood, whilst a pulmonary vessel must have been severed in order to account for the effusion of blood, which rapidly attained its maximum and suddenly stopped. The hæmorrhage is supposed to have ceased when the blood filled the pleural cavity to the level of the pleural pulmonary wound. At this moment the wound was closed by sutures and healed by first intention without any untoward symptom. The wound was received on the 15th of June, and the man left the hospital on the 9th of July.

*Laparotomy.*—X, 63 years of age, had been troubled for twenty years with an inguinal hernia, which was easily replaced.

It was replaced on the 9th of December, but from this date the usual symptoms of intestinal obstruction set in and persisted, notwithstanding all the remedies employed. M. Terrier, supposing there was an internal hernia or invagination which he could not locate, determined to open the abdomen in the median line. At the final examination previous to the operation there was perceived a spot less yielding than the adjacent parts. This was supposed to be the seat of constriction. After making an incision immediately below the umbilicus, eight centimeters in length, and after the opening of the peritoneum, there was found an hernia situated in the anterior abdominal wall where the unyielding spot was discovered. Reduction being effected, the wound was closed and completely healed in ten days. It is thus being daily demonstrated that the wounding of the serous membranes is not necessarily attended with so much danger as was formerly supposed.

